# From Angiopoietin-2 to basic chemistry: differential effects of statins on endothelial homeostasis in ARDS

**DOI:** 10.1186/s13054-023-04460-3

**Published:** 2023-05-12

**Authors:** Daniel A. Hofmaenner, Sascha David

**Affiliations:** Institute of Intensive Care Medicine, University Hospital Zurich, Raemistrasse 100, CH-8091 Zurich, Switzerland

Dear Editor

With great interest, we have read and appreciated the recent article by Pienkos et al., published in the *Journal* [1].

The authors present a secondary analysis of two large randomized trials [2, 3] investigating the effect of statin therapy in patients with the acute respiratory distress syndrome (ARDS), taking initial total cholesterol levels into account. As a reminder, both original trials were negative with regard to hard clinical endpoints. In this additional post-hoc analysis, the authors now describe differential treatment effects dependent on the initial total cholesterol levels and the statin evaluated. Stratification for baseline cholesterol levels revealed higher disease burden in the lowest cholesterol quartiles and opposing effects between the two statins analyzed. Despite all limitations with such a post-hoc analysis, it is impressive that simvastatin showed a reduced patient mortality, but rosuvastatin was associated with harm [1]. In fact, low cholesterol levels have long been recognized to be associated with worse patient outcomes in sepsis and ARDS [4]. While low cholesterol levels have been shown to be prognostic, the role of statins with their mysterious pleiotropic effects in critically ill patients with ARDS appears less consistent. We thus would like to address some points that merit further discussion.

First, ARDS is a syndrome known to be associated with diffuse alveolar damage and capillary leakage of the pulmonary vasculature. Besides direct injury to epithelial structures, it is the endothelium itself that holds a key position in the pathophysiology and the progression of the syndrome. In this context, elevated Angiopoietin-2 (Angpt-2), an antagonistic ligand of the endothelial Tie2 receptor, has been implicated to be a causal contributor to endothelial activation and thereby to the pathogenesis of ARDS. In animal and cell culture models, Angpt-2 deactivates the protective endothelial tyrosine kinase Tie2 leading to increased endothelial permeability, massive recruitment of inflammatory cells and even apoptosis. From a translational research point of view, it might thus be worth to consider targeting this injurious vascular permeability factor in ARDS. Interestingly, we found in an unbiased FDA-library screening of almost 900 approved compounds, that two lipophilic statins (i.e., Simvastatin and Lovastatin) revealed a potential effect on Angpt-2 [5]. In a set of experiments both in vitro and in vivo, we could demonstrate a strong effect of Simvastatin on Angpt-2 downregulation, thereby promoting a pleiotropic survival benefit in an experimental sepsis model. Of note, the effect on Angpt-2 was strongest with Simvastatin but also detectable with other tested lipophilic statins, whereas Rosuvastatin (being a hydrophilic drug) had absolutely no effect on Angpt-2 biosynthesis (own unpublished data).

While differences in pharmacokinetics such as absorption and hepatic metabolism among different statins are well-known, mechanistic data are lacking as to whether hydrophilic or hydrophobic properties of statins might be causally involved in the pathogenesis of *capillary leakage* on a local level. It only can be speculated that different statins might exhibit differing capacities to diffuse through injured, permeable endothelia.

We thus would like to highlight that the phenomenon of differential effects of hydrophilic vs lipophilic statins on Angpt-2 could very well explain why simvastatin but not rosuvastatin was associated with clinical benefits in the article by Pienkos et al. [1]. If we would all agree in believing for a second that indeed Angpt-2 is a relevant target of lipophilic statins, this could open new horizons for clever future trial stratifications for a targeted treatment strategy. In the future, sophisticated translational research implementing this approach could mark another example of bench-to-bedside research improving patient outcomes with sepsis and ARDS.

## Data Availability

Not applicable.
